# Preschool experiences and home learning environments of migrant children in urban China

**DOI:** 10.3389/fpubh.2023.1086439

**Published:** 2023-02-13

**Authors:** Jing Gong, Nirmala Rao

**Affiliations:** Faculty of Education, The University of Hong Kong, Hong Kong, Hong Kong SAR, China

**Keywords:** educational inequality, preschool education, home learning environments, migrant children, China

## Abstract

Internal migration and urban expansion, hallmarks of rapid urbanization in China, have led to an increasing number of children with diverse backgrounds in cities. Cities now include migrants from rural and urban areas, and children from “urban villages” in addition to “urban locals”. Parents of young children who migrate from rural to urban areas leave their children behind in rural areas (“left-behind” children) or take them along with them. In recent years, increasing parental migration from one urban area to another has also led to children being “left-behind” in urban areas. This study examined the preschool experiences and home learning environments of rural-origin migrants, urban-origin migrants, and rural-origin locals in comparison to urban locals, leveraging data from the nationally representative China Family Panel Studies (2012–2018) with 2,446 3- to 5-year-olds residing in urban areas. Regression model results indicated that children living in cities who held a rural household registration certificate (*hukou*) were less likely to attend publically funded preschools and experienced less stimulating home learning environments than urban local children. After controlling for family characteristics (i) rural-origin locals remained less likely to participate in preschool and experienced fewer home learning activities than urban locals; and (ii) there were no differences in preschool experiences and home learning environments between rural-origin migrants and urban locals. Mediation analyses suggested that the relation between *hukou* status and the home learning environment was mediated by parental absence. Implications of the findings are discussed.

## 1. Introduction

Since 2008, over 50% of the world's population has lived in urban areas (1) and increasing numbers of children will grow up in towns and cities. Along with rapid urbanization, poverty and inequality are also increasing across the world's cities. For instance, an estimated 300 million migrant children live in urban slums and experience polluted, insecure living environments or educational exclusion (2, 3). China is one of the most rapidly urbanizing countries in the world. The large-scale rural-to-urban migration and the fast-growing metropolises have led to an increasingly urban population. The urbanization rate in China soared from 49.68% in 2010 to 63.89% in 2020 (4). The proportion of preschool-aged migrants has also increased in the recent decade (5). About 5.9 million 3- to 5-year-olds migrated with their parents from one part of China to another in 2015 (6). Therefore, there is a need for research on children's early experiences in the context of urbanization to inform evidence-based policies and programs.

A distinction is made between internal migration, which occurs within a country, and external or international migration. National household registration systems such as the *hukou* in China and the *ho khau* in Vietnam present institutional barriers for internal migrants (7). Beyond East Asia, unfavorable integration policies have also adversely impacted the educational outcomes of international migrants who have moved to western countries (8).

This paper focuses on internal migration in China. The urban-rural dual structure is a unique feature of Chinese society, with the household registration (*hukou*) system at its core. The *hukou* system denotes each newborn as having either an agricultural (rural) *hukou* or a non-agricultural (urban) *hukou*, depending on their parents' *hukou* status and location (9). One's *hukou* status determines where one can access public services such as healthcare and education. Urban residents who have a rural *hukou* are not entitled to access all the same public services in urban areas as local urban *hukou* holders (5, 9). Therefore, the *hukou* system has been widely recognized as an obstacle to the adaptation of new migrants in China. Without an urban *hukou*, rural-to-urban migrant children experience institutional barriers, social exclusion, and have limited access to educational opportunities. Indeed, research has demonstrated that rural-origin migrants have poorer academic performance and more socio-emotional problems than urban local children (10, 11). In order to provide appropriate support to migrant children and families, it is important to understand the reasons for disparities in early learning and development between migrant and local children.

A large number of rural areas on the outskirts of cities have been converted to new urban districts in China, and these are referred to as “urban villages”. Rural residents in urban villages have been re-classified as urban residents and are referred to as rural-origin locals in this study. These urban villages are transitioning into becoming urban with an apparent mixture of rural and urban social norms, and often provide residential space for local landless farmers and provide low-cost housing for migrants (12). While rural-to-urban migrant children have received policy and research attention, the educational opportunities for local children in the urban villages are little understood.

Disparities within and across cities in China have widened due to internal migration and urban expansion, motivating migration from small towns or cities to better resourced urban areas. The number of urban-to-urban migrant children and urban children being left-behind by their parents has increased. This warrants concern as it has been demonstrated that urban left-behind children are also disadvantaged in academic performance and mental health (13, 14) compared to urban local children. However, a limitation of the existing studies in China is the tendency to exclusively focus on rural-to-urban migrant children, leading to a paucity of research on the impact of new internal migration trends (15), such as urban-to-urban migration and the impact of urban expansion on early learning and development.

Against this background, we categorized children into four distinct groups living in urban China based on their *hukou* and migrant status: rural-origin migrants (ROM), urban-origin migrants (UOM), rural-origin locals (ROL), and urban locals (UL) (see Table 1). The first two letters in the acronym denote *hukou* status. The last letter indicates whether the child is local or a migrant. Substantial evidence indicates that the quality of center- and home-based learning experiences during early childhood are positively associated with child and adulthood outcomes (16, 17). Leveraging a nationally representative sample, this study compares the early learning experiences of rural-origin migrant children, urban-origin migrants, and rural-origin locals, with urban local children. Therefore, this study extends and complements past studies that have tended to focus exclusively on rural-to-urban migrant children. Additionally, we examine the relation among early learning experiences, *hukou*, and parental absence in urban China.

**Table 1:** Terms used to describe children in urban China.

	**Child**	**Current residence**	**Household registration**	**Migrant**
1.	Rural-origin migrants (ROM)	Urban	Rural	Yes
2.	Urban-origin migrants (UOM)		Urban	Yes
3.	Rural-origin locals (ROL)		Rural	No
4.	Urban locals (UL)		Urban	No

## 2. Preschool experiences in China

In China, the term preschool education is used to denote services provided to children aged 3 to 6, either in stand-alone preschools known as kindergartens or in a one-year pre-primary class located in a primary school (18). In kindergartens, children are divided into classes based on their age, and there are three “grade” levels. Children aged 3 to 4 are in the junior class; those aged 4 to 5 years are in the middle class; and those ranging in age from 5 to 6 years are in the senior class. Children who live in rural areas where there are no stand-alone kindergartens may attend a pre-primary class for 1 year before they start Primary 1. The one-year program aims to prepare children, with no experience in a stand-one kindergarten, for the transition to primary school. We use the term preschool to refer to education services for children aged 3 to 6 years, provided in both stand-alone kindergartens and in pre-primary classes. Furthermore, kindergartens in China can be classified as public or private depending on their funding source. Compared to private preschools, publically funded preschools (hereafter, public preschools) tend to have more stable funding from the government, are better regulated, and provide greater job stability and benefits. They are usually considered to charge reasonable fees and to be of satisfactory quality (19, 20).

Many studies have documented urban-rural and coastal-inland disparities in educational resources and children's preschool experiences (21–23). In 2010, the Chinese government launched the National Medium and Long-term Education Reform and Development Plan (2010–2020), which prioritized promoting access to preschool education in rural and less-developed central and western regions in China. With a series of preschool education policies, regional disparities in preschool attendance have narrowed (22, 24). However, regional disparities in preschool quality remain, and preschool teachers in rural areas are less qualified than their urban counterparts (24). Hu and colleagues highlighted a growing concern about the high proportion of preschool programs operated by private providers and the widening gap in the quality of private rural programs compared to public preschools or those in urban areas (25). It should be noted that while urban-rural disparities in preschool education have been documented, disparities in preschool education within and across cities in China are less well understood.

In most low- and middle-income countries, participation in preschool education is strongly related to regions of residence and family socioeconomic status (SES) (26, 27). Children in urban areas or wealthier households are more likely to participate in early childhood education programs than those in rural areas or from poor households. Studies in China have also highlighted rural and urban gaps in preschool attendance, and family SES is positively related to preschool attendance (22). Past work on the preschool attendance of migrant children has consistently reported that the preschool attendance rates for migrant children and urban local children are comparable and higher than those for children living in rural areas (23, 28). This finding is in line with the recent data from the Ministry of Education and the National Bureau of Statistics (4). In 2020, the preschool enrollment rate for migrant children aged 3 to 5 was 86.1%, slightly higher than the overall national preschool enrollment rate of 85.2%. This may be because most migrant parents are working and rely on preschools for child care.

Nevertheless, the *hukou* may hinder migrant children's access to quality preschool education. Migrant children were less likely to attend public preschools than urban local children. About 29% of migrant children attended public preschools (29), which was 21.7% lower than the national average public preschool enrollment rate (30). Besides institutional barriers, the distribution of educational resources also affects preschool learning opportunities. For example, due to the higher supply of public preschools in eastern China, migrant children who moved to eastern China had the highest rate of attendance rate in public kindergartens, followed by those who migrated to western and central China, respectively (23).

There are several reasons for the difference in rates of access to public preschools between migrant and local children in urban China (31, 32). Most migrant families live in remote urban villages because of the relatively high costs of housing in cities (12). There is a lower supply of public preschools in urban villages and children with a local *hukou* are given enrollment priority. Migrant children are only likely to be admitted to public preschools if vacancies arise (31). Their parents are less familiar with the process and report difficulties in completing the complex steps to secure admission to public preschools. Furthermore, as migrant families are usually economically disadvantaged, they typically resort to sending their children to low-cost private preschools, which may be of low quality.

The Seventh National Population Census revealed new trends of internal migration in China with a marked increase in the urban-to-urban migrant population (4, 15). Yang and Xie (23) compared the preschool experiences of urban-origin migrants and rural-origin migrants drawing on the 2013 National Migrant Dynamic Monitoring Survey. They found no significant difference in their general preschool enrollment and that a higher percentage of urban-origin migrants accessed public preschools than rural-origin migrants. This finding suggests that urban-origin migrants may experience few institutional barriers and benefit from better educational resources in cities.

## 3. Home learning environments in China

Home learning environments (HLEs) refer to caregiver-child interactions that occur regularly at home and through which children acquire knowledge and skills. Along with national economic development and increased levels of educational attainment, parents in China have invested more time and money to support the learning and development of their young children. Nevertheless, regional and urban-rural disparities in HLEs remain significant. Children in urban areas and in more economically developed regions tend to receive more learning stimulation than those in rural and less developed regions (33). Furthermore, as parents in urban China are exposed to the influences of globalization, their parenting practices differ from those in rural areas. Child-rearing practices of urban parents reflect both traditional Chinese values and western notions of child-rearing (34).

Higher SES is associated with more stimulating HLEs (33, 35–37). When family income levels are similar, caregivers with higher levels of education are more likely to provide enriching HLEs to their children than parents with lower levels of education (38). Additionally, research has uncovered an indirect relation between caregivers' psychological distress and learning stimulation in low-income families. Caregivers in low-income families are more likely to experience financial hardship than other families. This increases their stress level and decreases their ability to be involved with their children (35). Besides family SES, the family structure and childcare arrangement also affect HLEs. Two-parent families are considered to be a more stable structure and beneficial for children's development, compared to single-parent families or multigenerational households (39, 40).

Studies have found that the home environment and parenting practices for migrant children and urban locals in early childhood vary (41, 42). Migrant children had a higher home chaos score than urban locals, indicating that they may experience a more disorganized, crowded, and noisy living environment (42). Qualitative studies have shown that migrant parents lack appropriate knowledge and skills and provide limited learning stimulation to their children (43, 44). For example, they tend to prioritize academic skills over socio-emotional development and overlook the important influence of home environments on the development of young children. Furthermore, many migrant caregivers are primarily engaged in jobs that demand long working hours and render relatively low wages, and thus may have less time and resources to promote their child's learning. A more recent study found that the childrearing beliefs and practices of migrant mothers in urban villages in mega-cities in China (Guangzhou and Shenzhen) approximated those of urban middle-class mothers (45).

Few studies have considered differences between rural-origin migrant and rural-origin local children. They share the characteristics of holding a rural *hukou* in urban areas, and many of them may live in urban villages. When urban villages underwent a transition from rural to urban social systems, both the extent of educational resources available and the parenting beliefs of original villagers did not change substantially during the urbanization process (46, 47). Within urban villages, local families tend to have greater financial resources than rural families. Most rural-origin local families are no longer involved in farming, and some of them have stable rental incomes (12). However, they possess relatively low levels of education, which may hinder their knowledge and ability to provide learning opportunities to their child at home (46, 47). Furthermore, Dai's study (48) found that to cope with the pressures of adapting to urban living and balancing work and family, landless farmers' families in urban villages adopted a three-generation childcare arrangement with grandparents being the primary caregiver for young children.

### 3.1. Parental absence and home learning environment

Parental absence is associated with less stimulating HLEs and may hinder children's development (49–51). According to the Chinese population census, the number of children not living with both parents in urban areas has increased from 3.10 million in 2000 to 28.26 million in 2015 (6). In previous studies, rural-origin migrant children and rural left-behind children have often been considered as two groups with different family migrant arrangements (52, 53). Evidence on the impact of parental absence on HLEs in China is mostly based on samples of rural children who have one or two migrant parents. Some children are left-behind with one parent, and others experience the absence of both parents. These studies have found that children living without both parents or with an absent mother receive relatively low levels of home-based stimulation (54, 55). However, children who migrated to cities may also experience parental absence. Only 45% of migrant children live with both parents, and 23.4% of migrant children live with non-family members (6). More research on the relation between parental absence and child development is needed so supportive policies and programs can be developed to promote family wellbeing.

## 4. Current study

**Figure 1:**
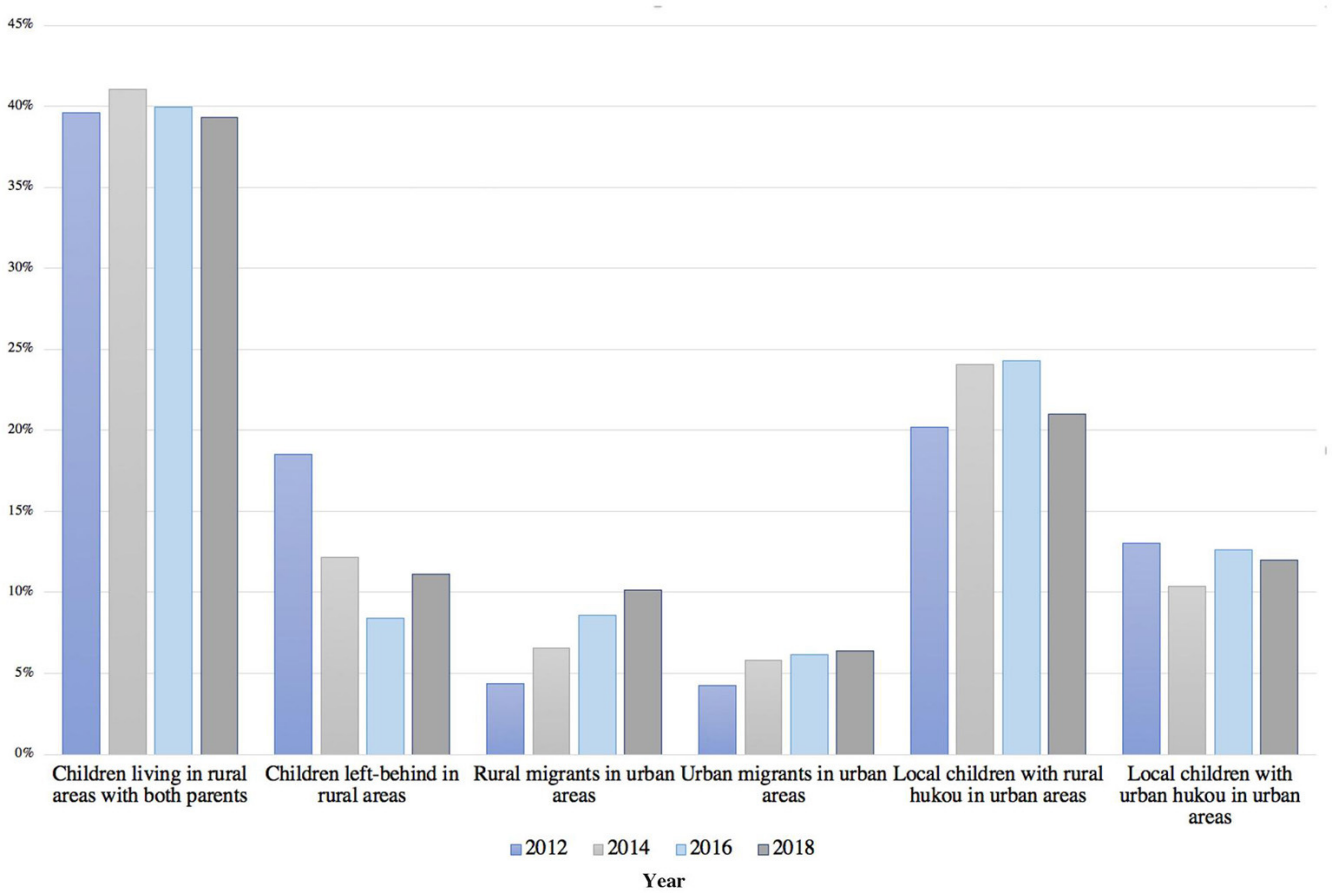
Living arrangements of 3- to 5-year-olds in China by year.

Figure 1 presents information on the area of residence of participants, aged 3 to 5, in the different waves of the China Family Panel Studies (CFPS). According to the authors' calculations, the percentage of children left-behind in rural areas by their parents declined from 2012 to 2018, and more parents took their children along with them to the cities. This may be because there are no suitable caregivers in the hometown and the perceived advantages of urban living. Additionally, the percentage of children residing in urban areas increased from 41.89% in 2012 to 49.56% in 2018. The percentage of children with urban *hukou* was 18.39% in 2018 and has remained stable over the years. The figures suggest that an increasing number of new urbanites and their children may experience challenges accessing public health, preschool education, and other publically funded social services in urban areas, which deserve further study. In this study, we aimed to extend previous research on urban-rural disparities to intra-urban disparities in early learning experiences in the context of urbanization, and four research questions (RQ) were addressed:

RQ1: Are there differences in the overall preschool attendance rate of rural-origin migrants, urban-origin migrants, rural-origin locals, and urban locals? Based on existing literature (4, 23), we hypothesize that the differences in the overall preschool attendance between rural-origin children and urban locals will not be significant.
RQ2: Are there differences in enrollment rates in public preschools among rural-origin migrants, urban-origin migrants, rural-origin locals, and urban locals? Given the *hukou* restriction and the unequal distribution of quality educational resources within urban areas, we hypothesized that a low portion of children with rural *hukou*, including rural-origin migrants and locals, would be enrolled in public preschools (23, 28). Furthermore, we hypothesized that holding a rural *hukou* will be associated with a lack to access to public preschool after controlling for family characteristics.
RQ3: Are there differences in home learning environments of rural-origin migrants, urban-origin migrants, rural-origin locals, and urban locals? As for home-based learning experiences, we hypothesized that rural-origin migrant children would experience less stimulating HLEs than urban locals (42). Furthermore, although families of rural-origin local children may have higher income, their parental education level was comparable with rural-origin migrant families (46, 47), which may result in a lower frequency of learning stimulation for rural-origin locals compared to urban local children.
RQ4: Does parental absence affect children's HLEs in urban China? What are the relations among the child's *hukou* status, parental absence, and home learning environments in urban China? We hypothesized that the negative impact of parental absence on HLEs in urban areas is similar to evidence based on rural children (54, 55). There is little empirical evidence for us to propose a hypothesis on factors that mediate the relation between *hukou* and HLEs, so this question is exploratory. We examined whether *hukou* status was associated with parental absence and, in turn, HLEs.

## 5. Methods

### 5.1. Data and sample

This study leveraged an unbalanced panel dataset from the CFPS, a large-scale, nationally representative longitudinal survey in China conducted by the Institute of Social Science Survey of Peking University. The baseline survey of CFPS was implemented in 2010, adopting a stratified three-stage sampling strategy. Approximately 14,960 households in 635 communities across 25 provinces were selected through this approach, representing 95% of the Chinese population (56). The CFPS includes rural and urban samples and permits analyses pertinent to the research questions. In the CFPS, five questionnaires concern information at community, household, and individual levels. One eligible family member provided basic information of the whole family, such as relationships of family members and socio-demographic data. Family members over 16 years answered the adult questionnaires. The guardian provided information on children under 15 years by answering the proxy child questionnaire. Core family members from the baseline sample were followed up every 2 years. At the time of writing, complete data from five waves of CFPS data (2010, 2012, 2014, 2016, and 2018) and some data from 2020 had been released.

This study focuses on the early learning experiences of preschool-aged children in urban China. As the information on the type of preschool was not available for the 2010 wave, we pooled cross-sectional data from four waves of the CFPS (2012, 2014, 2016, and 2018). We restricted our sample to children aged 3–5 years, living in urban areas, and with registered *hukou* status. The CFPS project team followed the Census Bureau's classification of rural or urban regions to code the child's current residence. The final sample consisted of 2,446 participants, including 591 children in 2012, 622 children in 2014, 721 children in 2016, and 512 children in 2018.

### 5.2. Variables

We considered three outcomes related to children's early learning experiences in this study: preschool attendance, the type of preschool program attended, and HLEs. Caregivers indicated whether the child was currently enrolled in a kindergarten or in a pre-primary class. Their responses were coded into a binary variable: 0 = no and 1 = yes. If the child was currently enrolled in a kindergarten or pre-primary class, a follow-up question was asked to identify the type of kindergarten or pre-primary class attended. The answer options were private and public. In the CFPS, a kindergarten or pre-primary class run by a private educational organization is considered private, regardless of whether it receives public assistance. Based on caregivers' responses on preschool enrollment status and the type of preschool, preschool experience was categorized into three categories (0 = not in school, 1 = private, and 2 = public).

The HLEs measure was constructed based on the caregiver's report of the frequency of four home learning activities: helping the child learn characters, reading to the child, taking the child out to play, and buying books for the child (α = 0.71). Responses for each home learning activity were measured on a 5-point Likert scale (1 = several times a year or less, 2 = once a month, 3 = 2 to 3 times a month, 4 = several times a week, 5 = every day). The mean score of four items was calculated to represent the HLEs for young children.

A categorical independent variable of the child group was created based on the child's *hukou* (urban = 0, rural = 1) and migration status (locals = 0, migrants = 1). Parents were asked about the location of the child's place of household registration. Options for this question included: the child's *hukou* is in the same village or residential community, in another village or residential community in this township, in another township in this county/city/district, in another county/city/district in this province, or in another province in mainland China. If the location of the village or residential community in which the child lived were different from the current place of household registration, the child would be considered a migrant child. Otherwise, the child was deemed to be a local child. As noted previously, we categorized children into four distinct groups: rural-origin migrants (ROM), urban-origin migrants (UOM), rural-origin locals (ROL), and urban locals (UL). In data analysis, the child group variable was dummy coded with UL as the reference group.

Parental absence was coded based on whether the child lived without one or both parents for more than 7 months a year (0 = no, 1 = yes). This indicator was constructed based on two questions. Caregivers were asked to report how long the child lived with his/her father and mother in the past year on a scale of 1 to 7 (Almost the entire year, 11, 8–10, 5–7, 2–4 months, and 1 month, almost never). If the answers to both questions were more than five months, the child would be considered as not experiencing parental absence in the last year. In contrast, the child was considered left-behind if the child lived without one or both parents for more than seven months a year.

We also considered several demographic characteristics as control variables, such as child age, gender, ethnic minority status, family SES, and region of residence in China. Age was measured in completed months. A binary child gender variable was included where 0 denoted male and 1 denoted female. A binary variable of minority status was created where 0 denoted the child was Han Chinese and 1 denoted that the child was from an ethnic minority group. A composite of five variables was used to designate family SES: household wealth, paternal and maternal education levels, and paternal and maternal employment status. The annual net family income per capita (past year) in Chinese yuan was adjusted to be comparable with the year 2010 by the CFPS project team, and we used the log-transformed value for analyses. Paternal and maternal education levels were determined by the highest level of education that the child's father/mother obtained, with higher scores showing a higher education level (1 = Illiterate/Semi-literate, 2 = Primary school, 3 = Junior High school, 4 = Senior High school, 5 = 3-year College, 6 = 4-year College, 7 = Master's degree or above). The data analyses treated paternal and maternal education levels as continuous variables. Paternal and maternal employment status were two coded as binary variables indicating whether the father or mother was employed at the time of the interview (0 = no, 1 = yes). The child's region of residence was determined from the caregiver report of the province in where the child lived. Provinces were classified into three categories representing the three economic regions in China (western China, central China, and eastern China). Three regions were recoded as two dummy variables, with western China being the reference group. In addition, we included a categorical variable of the time point of survey (2012, 2014, 2016, and 2018 waves) to control the differences across waves. Four waves were recoded as three dummy variables, with 2012 being the reference group.

### 5.3. Data analysis plan

Before answering the first research question, we describe trends in the types of preschool programs that children attend and HLEs in the four groups of children living in urban areas from 2012 to 2018. In addition, one-way ANOVAs or chi-square tests were applied to compare demographic characteristics and early learning experiences among the four groups of children. To answer RQ 1 to 3, we ran two regression models for each dependent variable before and after controlling covariates. The categorical independent variable of the four child groups was dummy coded with urban locals as the reference group. In the first model, we only included child characteristics and waves as control variables. In the second model, family characteristics were added as control variables. The overall preschool attendance was the dependent variable for RQ1. Binary logistic regression was used to examine the relation between the child group and preschool attendance. The type of preschool programs was the dependent variable for RQ2. Multinomial logistic regression was used to investigate the relation between the child group and the type of preschool program attended. HLEs was the dependent variable for RQ3. Ordinary Least Squares (OLS) linear regression was used for HLEs. To ensure that observations were independent in the analysis, we only used data from the most recent wave for children interviewed in multiple waves. We adopted multiple imputations by chained equation (MICE) to deal with the missing data (57). Missing data on covariates range from 0 to 13.97% on each variable. Missing data were imputed using MICE to create thirty imputed data sets. Continuous variables with missing data were imputed by predictive mean matching, and categorical variables with missing data were imputed using logistic models.

**Figure 2:**
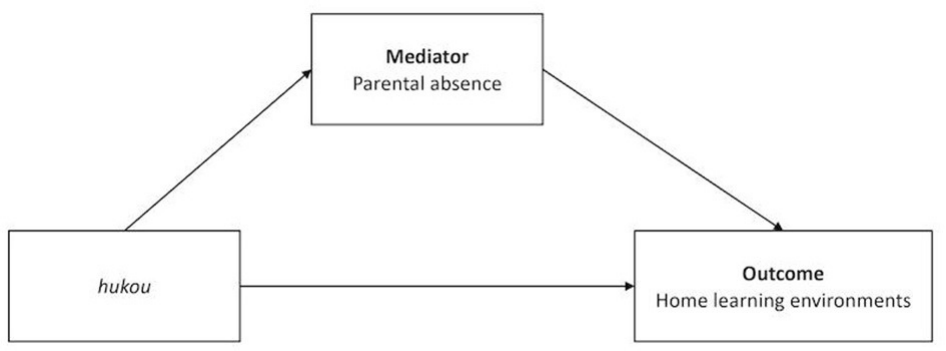
Conceptual path diagram of mediation model.

To answer RQ4, we examined the relation between parental absence and three outcomes of early learning experiences separately using regression analyses. Lastly, a path model was specified to test the mediating mechanisms from *hukou* to HLEs through parental absence (see Figure 2). Control variables for the mediation model included the child's age in months, gender, and waves of data collection. Based on Hu and Bentler's recommendations (58), several fit indices were used to evaluate the model fit. Acceptable model fits were defined by a comparative fit index (CFI) ≥0.90, standardized root mean residual (SRMR) ≤ 0.08, and root mean square error of approximation (RMSEA) ≤ 0.08. The 95% bootstrap confidence intervals estimates using the percentile method were used to test the significance of direct, indirect, and total effects in the path model. The effect can be supported if the 95% confidence interval of the 1,000 bootstrap estimates did not include zero. Missing data were handled by full information maximum likelihood estimation approach for the mediation analysis. All analyses in this study were conducted using Stata version 14 (StataCorp).

## 6. Results

### 6.1. Descriptive information on demographic characteristics and early learning experiences

The demographic characteristics of the overall and subgroup samples and the result of bivariate analyses are presented in Table 2. The sample consists of 374 ROM (15.29%), 289 UOM (11.82%), 1,161 ROL (47.47%), and 622 UL (25.43%). The average age of the sample was 51.43 months (*SD* = 9.19), and the percentage of girls was 47.02%. Child age and gender did not differ across the four groups. Overall, 7.83% of the sample were ethnic minorities. A higher percentage of ROL were ethnic minorities than other groups of children (10.71%; χ^2^ = 26.88, *p* < 0.001). As for family characteristics, there were significant differences among the four groups in family income, maternal and paternal education levels, and maternal and paternal employment status. ROL had the lowest household income and paternal and maternal level education among the four groups. The average paternal and maternal education levels for ROL were lower than junior high school. In addition, compared to other groups, a lower percentage of ROL's parents currently had a job. The household income and paternal and maternal education levels for ROM children were slightly higher than ROL but lower than the other two groups of urban-origin children. Fathers of ROM children reported the highest employment rate, while the employment rate of mothers of ROM was similar to ROL and lower than the two urban-origin groups. UOM were the most advantaged in family SES among the four groups, with the highest household income level and paternal and maternal education levels. Also, over 80% of mothers of UOM reported currently having a job, which was higher than ROL (65.71%) and ROM (66.67%). Furthermore, there was a difference in the region where children resided (χ^2^ = 87.22, *p* < 0.001). Specifically, a higher percentage of ROL (28.34%) lived in western China than ROM (20.86%), UOM (18.69%), and UL (13.83%), and a lower proportion of them was in eastern China.

**Table 2:** Sample characteristics and bivariate analyses.

		**Overall sample**		**Urban locals**		**Rural-origin locals**		**Urban-origin migrants**		**Rural-origin migrants**			
		**Mean/%**	**SD**	**Mean/%**	**SD**	**Mean/%**	**SD**	**Mean/%**	**SD**	**Mean/%**	**SD**	χ^2^***/F*** **(*****df*** = **3)**	***p***
Child age (months)		51.43	(9.19)	51.47	(9.13)	51.88	(9.19)	50.92	(9.13)	50.39	(9.26)	2.41	
Female		47.02		46.62		46.94		48.10		47.06		0.18	
Minority		7.83		4.84		10.71		4.20		6.68		26.88	^***^
Ln (annual income)		9.04	(1.53)	9.41	(1.35)	8.65	(1.50)	9.62	(1.54)	9.21	(1.56)	53.07	^***^
Paternal education		3.52	(1.37)	4.25	(1.37)	2.90	(1.06)	4.51	(1.42)	3.46	(1.17)	234.76	^***^
Maternal education		3.48	(1.35)	4.14	(1.36)	2.91	(1.05)	4.61	(1.35)	3.32	(1.12)	238.08	^***^
Paternal employment status		91.16		91.84		88.61		94.86		95.02		18.55	^***^
Maternal employment status		69.28		72.31		65.71		80.86		66.67		25.86	^***^
Place of residence	Western	22.36		13.83		28.34		18.69		20.86		87.22	^***^
	Central	29.84		31.67		32.39		22.49		24.60			
	Eastern	47.79		54.50		39.28		58.82		54.55			
Year	2012	24.16		29.58		24.55		20.76		16.58		37.32	^***^
	2014	25.43		22.19		27.56		26.64		23.26			
	2016	29.48		28.30		29.20		29.76		32.09			
	2018	20.93		19.94		18.69		22.84		28.07			
Preschool attendance		72.00		76.53		68.82		75.09		71.93		13.51	^**^
Type of preschool	No	30.27		25.57		33.49		27.91		29.75		43.67	^***^
	Private preschool	43.35		44.31		43.76		32.95		48.16			
	Public preschool	26.38		30.12		22.76		39.15		22.10			
HLEs		2.86	(0.95)	3.21	(0.81)	2.54	(0.97)	3.29	(0.78)	2.96	(0.89)	103.49	^***^
Reading		3.17	(1.44)	3.66	(1.31)	2.73	(1.44)	3.76	(1.21)	3.26	(1.39)	82.03	^***^
Learning characters		3.09	(1.40)	3.39	(1.29)	2.78	(1.42)	3.48	(1.27)	3.26	(1.38)	38.73	^***^
Outings		3.14	(1.30)	3.52	(1.10)	2.77	(1.37)	3.64	(1.03)	3.27	(1.21)	71.01	^***^
Buying books		2.05	(1.03)	2.26	(1.02)	1.87	(1.00)	2.29	(0.98)	2.05	(1.05)	25.60	^***^
Parental absence		19.92		13.37		25.47		12.50		19.30		49.16	^***^
*N* of Observation		2,446		622		1,161		289		374			

SD, Standard deviation. P-values are calculated with the chi-square (χ^2^) or ANOVA tests depending on the type of variable. The asterisks are used to denote a statistically significant result in the chi-square or ANOVA tests, ^***^p < 0.001; ^**^p < 0.01; ^*^p < 0.05.

As shown in Table 2, the overall preschool attendance rate for the total sample was 72.00%, with ROL (68.82%) being slightly less likely to attend preschool than the other three groups. Approximately 26.38% of children in the total sample attended public preschool programs. The percentage of children accessing public preschool was lower for ROM (22.10%) and ROL (22.76%) than for UL (30.12%) and UOM (39.15%). The average score of HLEs for the total sample was 2.86 (*SD* = 0.95), indicating that urban parents provide learning stimulation to their children on a monthly basis. The score of HLEs was the lowest for ROL (*M* = 2.54, *SD* = 0.97) and followed by ROM (*M* = 2.96, *SD* = 0.89).

### 6.2. Trends in preschool experiences and HLEs

**Figure 3:**
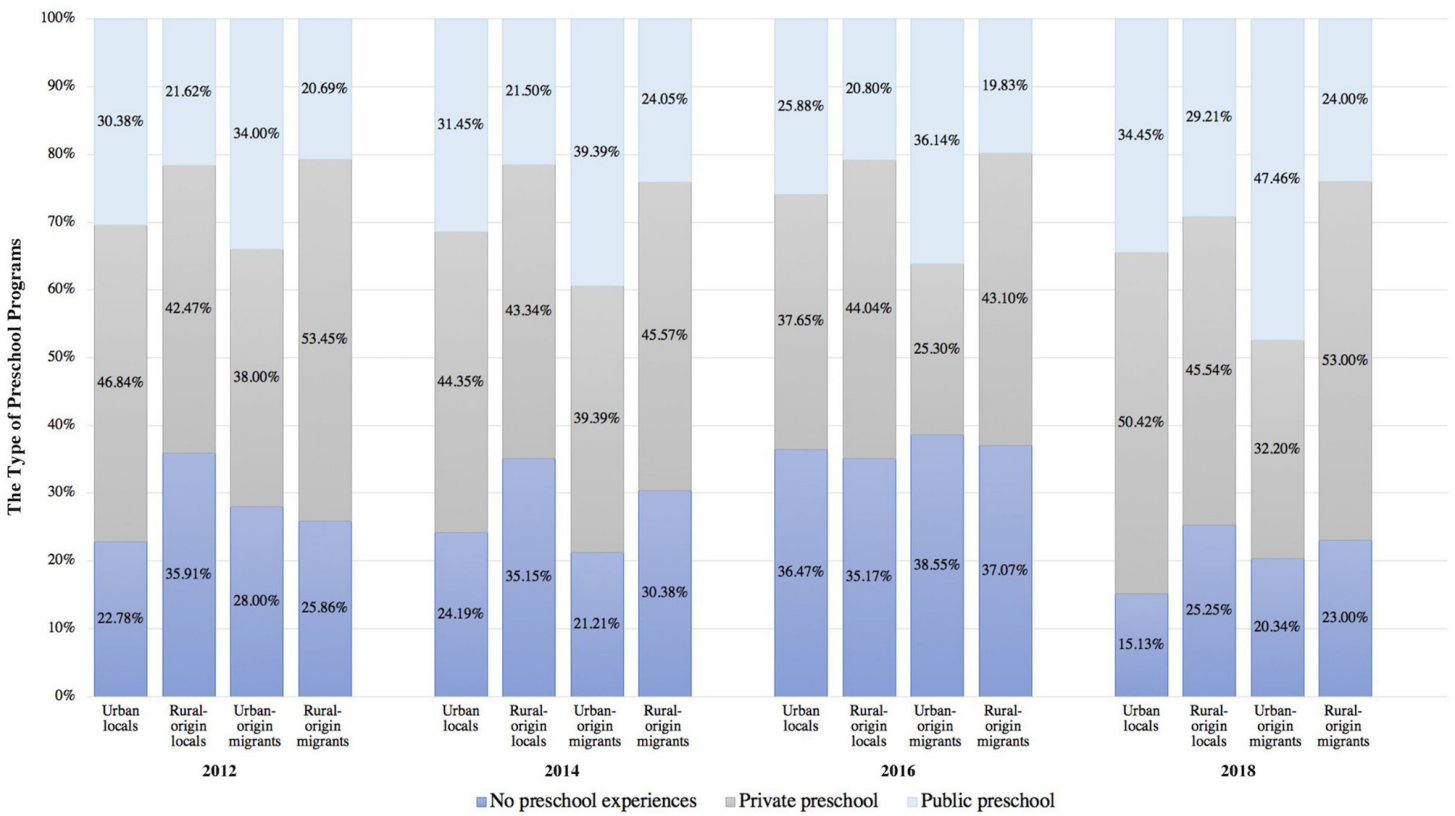
The type of preschool program urban children enrolled in by year.

The percentages of attendance in different types of preschool programs for the four groups of children from 2012 to 2018 are presented in Figure 3. There was an overall increasing trend of preschool attendance and access to public preschools for all four groups of children, despite an unexpected increase of no preschool experiences in 2016. UOM had the highest rate of attendance in public preschools among all groups at all four time points, and the gap between rural-origin and urban-origin children in accessing public preschools persisted across the years.

**Figure 4:**
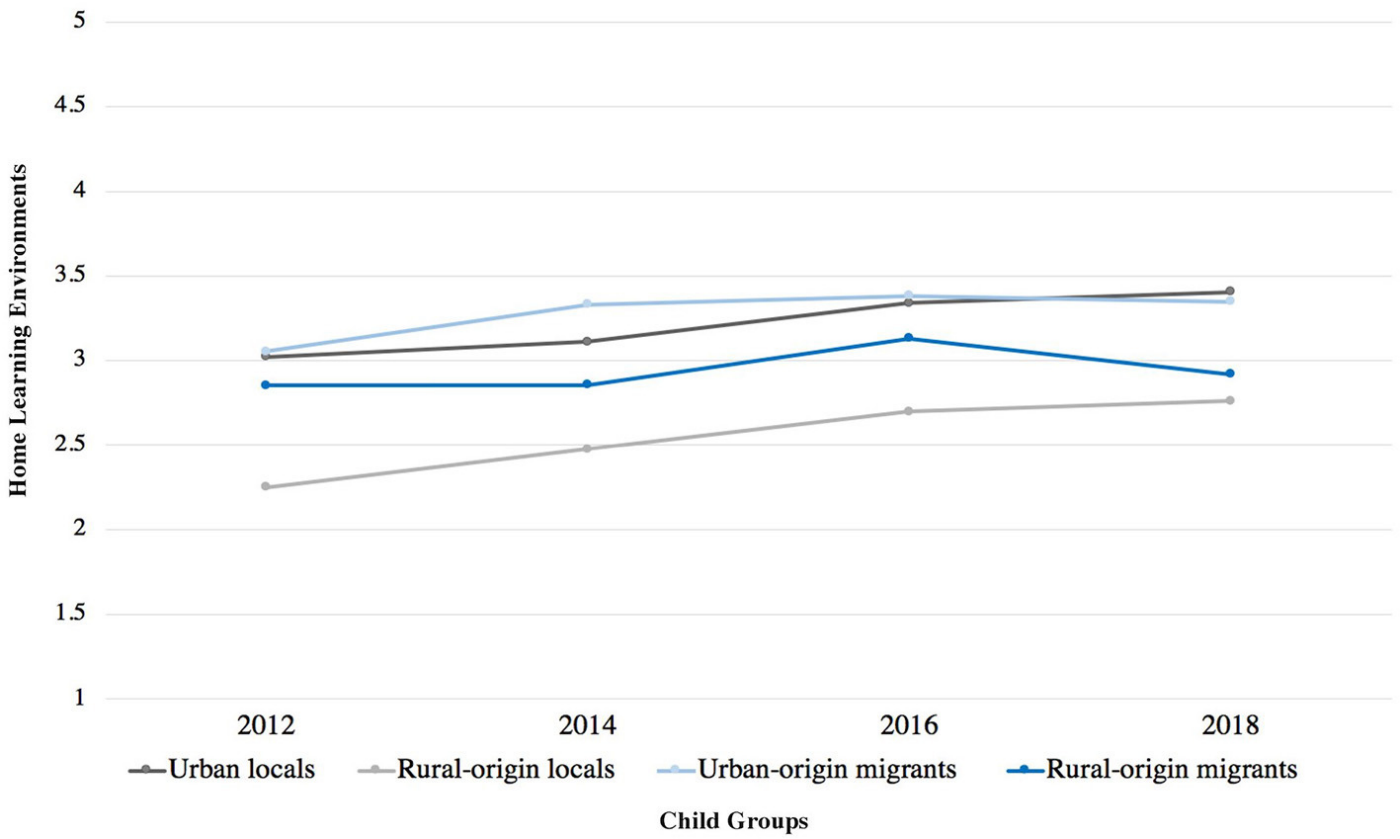
Home learning environments of urban children by year.

Figure 4 shows the mean score of HLEs among four groups of children across waves. There was an increasing trend in the frequency of HLEs for all groups of children except for a slight decrease from 2016 to 2018 for ROM. In 2018, ROM children (*M* = 2.92) and ROL (*M* = 2.76) experienced less frequent home learning stimulation compared to urban-origin children (UOM: *M* = 3.35; UL: *M* = 3.41), a gap evident over the years.

### 6.3. Results of regression analyses

#### 6.3.1. RQ1: Differences in the overall preschool attendance across groups

Table 3 presents the result of regression analyses for preschool attendance and the type of preschool program attended. Controlling for child age, gender, and waves, the preschool attendance rate for ROL was significantly lower than UL (Model 1; *OR* = 0.53, *SE* = 0.09, *p* < 0.001), and there was no difference in preschool attendance between ROM and UL. After adding family characteristics as control variables, ROL remained less likely to attend preschool compared to UL (Model 2; *OR* = 0.61, *SE* = 0.12, *p* < 0.01).

**Table 3:** Regression models for preschool attendance and the type of preschool programs.

	**Preschool attendance**						**The type of preschool programs**											
	**Model 1** **(*****N*** = **1,876)**			**Model 2** **(*****N*** = **1,876)**			**Model 3** **(*****N*** = **1,876)**						**Model 4** **(*****N*** = **1,876)**					
							**Private preschool**			**Public preschool**			**Private preschool**			**Public preschool**		
	**OR**	**SE**	***p***	**OR**	**SE**	***p***	**RRR**	**SE**	***p***	**RRR**	**SE**	***p***	**RRR**	**SE**	***p***	**RRR**	**SE**	***p***
**Child groups (ref: Urban locals)**																		
Rural-origin locals	0.53	(0.09)	^***^	0.61	(0.12)	^**^	0.62	(0.11)	^*^	0.51	(0.10)	^**^	0.67	(0.14)		0.64	(0.15)	^*^
Urban-origin migrants	1.04	(0.26)		1.12	(0.29)		0.84	(0.22)		1.50	(0.40)		0.92	(0.25)		1.54	(0.42)	
Rural-origin migrants	0.73	(0.16)		0.83	(0.19)		0.90	(0.20)		0.60	(0.15)	^*^	0.99	(0.24)		0.70	(0.19)	
Child age (months)	1.15	(0.01)	^***^	1.15	(0.01)	^***^	1.14	(0.01)	^***^	1.17	(0.01)	^***^	1.14	(0.01)	^***^	1.18	(0.01)	^***^
Female (ref: Male)	0.83	(0.11)		0.87	(0.12)		0.86	(0.12)		0.80	(0.13)		0.91	(0.14)		0.84	(0.14)	
**Year (ref: 2012)**																		
2014	0.87	(0.18)		0.87	(0.20)		0.87	(0.19)		0.94	(0.23)		0.89	(0.21)		0.93	(0.24)	
2016	0.68	(0.14)		0.62	(0.13)	^*^	0.75	(0.16)		0.80	(0.19)		0.72	(0.17)		0.72	(0.18)	
2018	0.99	(0.20)		0.86	(0.19)		1.00	(0.22)		1.18	(0.28)		0.92	(0.22)		0.97	(0.26)	
Parental absence				0.77	(0.13)								0.72	(0.13)		0.82	(0.16)	
Minority (ref: Han)				0.40	(0.09)	^***^							0.34	(0.09)	^***^	0.47	(0.13)	^**^
Ln (annual income)				0.95	(0.05)								0.96	(0.05)		0.96	(0.05)	
Paternal education				0.95	(0.07)								0.92	(0.07)		1.05	(0.09)	
Maternal education				1.13	(0.09)								1.10	(0.09)		1.10	(0.10)	
Paternal employment status				1.27	(0.37)								1.17	(0.34)		1.24	(0.40)	
Maternal employment status				1.37	(0.22)	^*^							1.35	(0.22)		1.37	(0.25)	
**Place of residence (ref: Western)**																		
Central				2.35	(0.50)	^***^							2.51	(0.55)	^***^	1.69	(0.40)	^*^
Eastern				0.88	(0.15)								0.82	(0.16)		0.83	(0.17)	
Constant	0.01	(0.00)		0.00	(0.00)		0.01	(0.00)		0.00	(0.00)		0.01	(0.00)		0.00	(0.00)	

OR, Odds ratio; RRR, Relative risk ratio; SE, Standard error; ^***^p < 0.001; ^**^p < 0.01; ^*^p < 0.05.

#### 6.3.2. RQ2: Differences in access to public preschools across groups

In Model 3, ROM was less likely to access public preschool than UL controlling for child age, gender, and waves (*RRR* = 0.60, *SE* = 0.15, *p* < 0.05). The likelihood of accessing private preschool and public preschool for ROL was significantly lower than UL (Private preschool: RRR = 0.62, SE = 0.11, *p* < 0.05; Public preschool: RRR = 0.51, SE = 0.10, *p* < 0.01). After adding family characteristics as control variables, there was no statistically significant difference in the type of preschool programs between ROM and UL. However, ROL remained less likely to access public preschools than UL (Model 4; *RRR* = 0.64, *SE* = 0.15, *p* < 0.05).

#### 6.3.3. RQ3: Differences in HLEs across groups

Table 4 presents the result of regression analyses for HLEs. ROM and ROL experienced a lower frequency of home learning activities than UL controlling for child age, gender and wave (Model 5; ROM: *B* = −0.34, *SE* = 0.07, *ES* = −0.13, *p* < 0.001; ROL: *B* = −0.67, *SE* = 0.05, *ES* = −0.35, *p* < 0.001). After adding family characteristics as control variables, ROM no longer displayed a statistical disadvantage in HLEs compared to UL. However, being a ROL was associated with experiencing less stimulating HLEs (Model 6; *B* = −0.30, *SE* = 0.05, *ES* = −0.16, *p* < 0.001).

**Table 4:** Regression models for home learning environments.

	**Home learning environments**							
	**Model 5 (*****N*** = **1,847)**				**Model 6 (*****N*** = **1,847)**			
	**B**	**SE**	**ES**	***p***	**B**	**SE**	**ES**	***p***
**Child groups (ref: Urban locals)**								
Rural-origin locals	−0.67	(0.05)	−0.35	^***^	−0.30	(0.05)	−0.16	^***^
Urban–origin migrants	0.04	(0.07)	0.01		−0.05	(0.07)	−0.02	
Rural–origin migrants	−0.34	(0.07)	−0.13	^***^	−0.12	(0.06)	−0.05	
Child age (months)	−0.01	(0.00)	−0.08	^***^	−0.01	(0.00)	−0.06	^**^
Female (ref: Male)	0.00	(0.04)	0.00		0.00	(0.04)	0.00	
**Year (ref: 2012)**								
2014	0.18	(0.06)	0.08	^**^	0.14	(0.06)	0.06	^*^
2016	0.43	(0.06)	0.20	^***^	0.28	(0.06)	0.13	^***^
2018	0.41	(0.06)	0.19	^***^	0.19	(0.06)	0.09	^**^
Parental absence					−0.15	(0.05)	−0.06	^**^
Minority (ref: Han)					−0.12	(0.08)	−0.03	
Ln (annual income)					0.03	(0.01)	0.05	^*^
Paternal education					0.10	(0.02)	0.14	^***^
Maternal education					0.15	(0.02)	0.22	^***^
Paternal employment status					0.24	(0.08)	0.07	^**^
Maternal employment status					−0.10	(0.05)	−0.05	^*^
**Place of residence (ref: Western)**								
Central					−0.08	(0.06)	−0.04	
Eastern					−0.02	(0.05)	−0.01	
Constant	3.46	(0.15)			2.02	(0.20)		
R-square	0.15				0.25			
Adjusted R-square	0.15				0.25			

B, Coefficient estimates; SE, Standard error; ES, Effect size; ^***^p < 0.001; ^**^p < 0.01; ^*^p < 0.05.

#### 6.3.4. RQ4: The relation between *hukou*, parental absence and HLEs

Table 2 shows the percentage of urban children in the four sub-groups who experienced parental absence. The percentage of experiencing the absence of one or both parents was the highest in the ROL group (25.47%), followed by ROM (19.30%). UOL (13.37%) and UOM (12.50%) were less likely to experience parental absence compared to children with rural *hukou* in urban areas. The result of the chi-square difference test showed that significant between-group differences in experiencing parental absence (χ^2^ = 49.16, *p* < 0.001).

In our sample, 46.30% of children experiencing parental absence were mainly looked after by their grandparents, while 18.88% of children with neither parent absent were looked after by grandparents. As the education level of grandparents tends to be lower than parents, grandparents sometimes lack knowledge of appropriate parenting practices (59), which may limit the amount of learning stimulation at home and hinder child development.

The results from the regression analyses of the relation between parental absence and three outcomes of early learning experiences are presented in Tables 3, 4. After adjusting for all covariates, urban children who experienced parental absence were less likely to have frequent home learning stimulation (Model 6; *B* = −0.15, *SE* = 0.05, *ES* = −0.06, *p* < 0.01) than other children. There was no significant relation between parental absence and children's preschool attendance and accessing public preschool programs.

The mediation analysis was performed to explore whether the *hukou*-based differences in HLEs were explained by parental absence, controlling for child age, gender and waves. Table 5 presents the model fit and direct and indirect effect estimates of the mediation model. Based on the fit index, the path model fit the data well. The indirect effect of the child's *hukou* status on HLEs mediated *via* parental absence was significant (β = −0.012, *B* = −0.023, *SE* = 0.008, 95% CI [−0.040, −0.008]), indicating parental absence explained the variation in HLEs by child *hukou* status. Having rural *hukou* increases the risk of experiencing parental absence (β = 0.151, *B* = 0.123, *SE* = 0.019, 95% CI [0.086, 0.159]), which in turn, decreases the frequency of home learning activities (β = −0.077, *B* = –0.186, *SE* = 0.052, 95% CI [−0.288, −0.083]). The direct effect of *hukou* on HLEs was also significant (β = −0.293, *B* = −0.571, *SE* = 0.043, 95% CI [−0.655, −0.487]). The ratio between the indirect effect and the total effect suggested that 3.80% of the disparity in HLEs between children with rural and urban *hukou* was mediated *via* parental absence.

**Table 5:** Estimates of the mediation analysis.

	**β**	**B**	**SE**	**CI lower**	**CI upper**	
**Direct path**						
*Hukou* → Parental absence	**0.151**	**0.123**	0.019	0.086	0.159	
Parental absence → HLEs	**−0.077**	**−0.186**	0.052	**–**0.288	**–**0.083	
*Hukou* → HLEs	**−0.293**	**−0.571**	0.043	**–**0.655	**–**0.487	
**The Effect from** ***hukou*** **to HLEs**						
The indirect effect of *hukou* on HLEs	**−0.012**	**−0.023**	0.008	**–**0.040	**–**0.008	
The total effect of *hukou* on HLEs	**−0.305**	**−0.594**	0.041	**–**0.666	**–**0.506	
**Model fit**	*χ2*	*df*	*p*	*CFI*	*RMSEA*	*SRMR*
	10.243	3	0.017	0.975	0.038 (0.014**–**0.064)	0.016
*N* of Observation	1,876					

Bolded results are significant at 0.05 level. Covariates included child age in months, gender and waves.

## 7. Discussion

Notwithstanding the declining birth rate in China, internal migration and urban expansion have led to an increasing number of preschool-aged children in urban areas. Institutional barriers such as the *hukou* system may lead to unequal access to educational and other resources in urban areas for young migrants. Quality early learning experiences lay the foundation for child development and school success (16, 17). This paper considers disparities in early learning experiences in urban areas in a rapidly urbanizing China. We leveraged nationally representative data from the CFPS (2012–2018) and identified four groups of children in urban China based on their *hukou* and migrant status: rural-origin migrants (ROM), rural-origin locals (ROL), urban-origin migrants (UOM), and urban locals (UL). Taken together, analyses to answer the four research questions indicated that there were disparities in preschool experiences and HLEs between rural-origin and urban-origin children in urban China and that these disparities persisted over time. Having a rural *hukou* was associated with less favorable early learning experiences. Furthermore, rural-origin children in cities experience parental absence, which, in turn, may reduce their HLEs. The disparities in early learning experiences in urban China are complex. This study contributes to a more nuanced understanding of the demographic characteristics and early learning experiences of different child groups in urban China and highlights areas for future research.

Although family SES differences across different groups of urban children are not the focus of our study, it is worth noting that the family income for ROL was the lowest among the four groups of urban children. Evidence from qualitative studies indicated that locals in urban villages are relatively wealthier than rural families as they often have stable rental incomes (12, 46). However, compared with urban families, they tend to be a disadvantaged group. UOM tend to be a more advantaged group in terms of family SES and early learning experiences. For example, parents who move from Beijing to Shanghai may be highly educated. Our study contributes to a deeper understanding of the family characteristics of different sub-groups of urban children.

This study found that all four groups of children generally received more stimulating early learning experiences with time. This finding is consistent with earlier evidence (22, 24, 33), and showcases the effectiveness of increased investment in ECE by the national government since 2010. However, barriers to migrants' access to public preschools remain. Su and Rao (33) argued that along with the overall increased family wealth and parental educational level in China, the child's HLEs improved. This pattern was observed for UOM, ROL, and UL, but the score of HLEs for ROM tended to flatten from 2012 to 2018. Since the implementation of the National New Urbanization Plan (2014–2020), the government has developed medium-sized cities and gradually lifted *hukou* restrictions with the goal of making it easier for migrants to settle in cities. However, an improvement in rural-origin migrant children's access to quality education and HLEs is not apparent, which indicates the need for child- and family-friendly policies for migrant workers with preschool-aged children in urban areas.

We found no significant differences in overall preschool attendance rates between ROM and UL but ROM were less likely than UL to access public preschool programs. It should be noted that the likelihood of ROL accessing preschool and public preschool programs was lower than UL after controlling for family characteristics. As discussed in previous qualitative research, there is a shortage of educational resources, especially quality preschool education in urbanizing villages (23). As noted earlier, ROM and ROL tend to live in urban villages, and there are barriers to enrolment in public preschools beyond *hukou* and family SES.

Furthermore, ROM and ROL received less stimulating learning experiences at home than UL. However, after controlling for their family characteristics, the HLEs for ROM and UL were similar. In our sample, parental educational levels of ROM were lower than UL, which may explain the variations in HLEs. At the same time, previous studies found that migrant parents hold as high expectations for their children as urban local parents. However, financial stress and unstable living and work environments might indirectly hinder parents from providing a nurturing and stimulating environment for their children (42, 60). Moreover, the finding showed that the differences in HLEs between ROL and UL remain even after controlling family SES, which indicates that different factors may influence HLEs in ROM and ROL. Motivation may be one possible explanation for this finding, as past studies have highlighted that migrating to cities is a family choice; migrants typically have a strong motivation for upward social mobility (12, 61). In contrast, ROL live in urban villages because of the expanding urban areas and may never leave their home villages (48). Further studies can provide a more nuanced understanding of the HLEs of ROL.

Caregivers of rural-origin children leave their home towns to seek better employment opportunities in urban areas. Consistent with previous research (54, 55), we found that parental absence is negatively associated with HLEs, regardless of whether children reside in urban or rural areas. Mediation analyses suggested that the relation between *hukou* status and the home learning environment was mediated by parental absence. Therefore, policy attention should be given to children with rural *hukou* in cities and those from less developed urban areas.

### 7.1. Implications

The findings of this study have implications for evidence-based early childhood policies and programs. First, data on enrolment in public preschools in cities should be disaggregated by *hukou* and migration status. The percentage of migrant children enrolled in compulsory education has been identified as a key indicator for tracking China's urbanization process in the 2014–2020 New Type Urbanization Plan (62). Data on the preschool enrollment rate for rural-origin migrant and local children should also be collected and monitored by the government regularly to index educational equity. Second, the Chinese government has prioritized preschool quality, and vulnerable children should have equitable access to high quality preschool programs. Increased effort should be exerted to ensure that preschools in urban villages are accessible and affordable to parents and that preschools provide high quality education. Effective social protection policies and practices, such as the conditional cash transfer programs in Brazil, have improved school attendance rates and school readiness for poor children in urban areas (3). Conditional cash transfer programs have been used in the Chinese context and their effectiveness in promoting preschool attendance may be explored (58, 63). Third, more support should be provided to vulnerable children from economically disadvantaged families. We found that family SES explained differences in early learning experiences between rural-origin children and urban locals. Therefore, policy and programs should not only target reducing institutional barriers for migrants but also include low-SES families. Fourth, more policy attention should be given to families in urban villages, as we found that children in urban villages experienced the least stimulating HLEs of all child groups. Parents in urban villages continue to be highly influenced by traditional Chinese parenting beliefs and practices (46, 47) which may be more relevant to those living in rural areas. Schools and communities can collaborate and support the acculturalization of underprivileged families in urban villages by providing them with information on age-appropriate child development and parenting practices. Fifth, given that a considerable number of children living in urban areas experience parental absence for over 6 months, support and education should be provided to caregivers, especially grandparents, in some urban villages. In short, within the Chinese context, it is important to provide tailored support for children based on their *hukou*, migration status, SES background, and place of residence.

### 7.2. Limitations and further research

Despite its contributions, our study has a few limitations. First, information about early learning experiences, be it HLEs or preschool experience, were reported by caregivers leading to common method bias. Second, the CFPS only measured the frequency of parental involvement in home learning activities. We have no information about the quality of the interactions. Third, we base our conclusions on the quality of preschools on the existing literature, which indicates that public preschools are of higher quality than private preschools. We did not measure preschool quality. Fourth, the cross-sectional nature of this study prohibits causal conclusions from the observed associations. Longitudinal data collected *via* direct assessment of preschool-aged children's home and center-based learning experiences are necessary.

Further research should provide a nuanced understanding of the reasons for disparities in early learning experiences between different groups of children. We found that institutional barriers affected children's early learning experiences. However, *hukou* restrictions vary across cities in China. Future studies can examine the association between the migrant destination and children's early learning experiences. Additional research can shed light on the experiences of children in urban villages and how it is affected by contextual characteristics such as the number of kindergartens and density of the migrant population.

## 8. Conclusions

This study highlighted the gaps in early learning experiences among rural-origin migrants, urban-origin migrants, rural-origin locals, and urban locals. Institutional and social barriers have precluded children with rural *hukou* in urban China from receiving quality preschool experiences and stimulating home learning activities. In addition, parental absence was associated with disparities in preschool-aged children's home learning experiences in cities. Our findings have implications for developing and implementing evidence-based policies and programs to promote educational equity. Rural-origin children, children from economically disadvantaged backgrounds, and those who experience parental absence have poorer early learning environments than their peers and require policy support. On a broader scale, our findings may apply to other developing countries experiencing rapid urbanization and that have large numbers of young migrant children. With sustainable urban planning and policy interventions, more families and children can enjoy the benefits of urbanization.

## Data availability statement

Publicly available datasets were analyzed in this study. This data can be found here: The datasets China Family Panel Studies (CFPS) for this study can be found in the Peking University Open Research Data Platform https://doi.org/10.18170/DVN/45LCSO.

## Ethics statement

The studies involving human participants were reviewed and approved by the Peking University Biomedical Ethics Review Committee. Written informed consent to participate in this study was provided by the participants' legal guardian/next of kin.

## Author contributions

JG and NR conceptualized and designed the study. JG performed the analyses and wrote the first draft with the support of NR. All authors critically reviewed this draft and approved the final draft for submission.
